# Feasibility of Duodenal Endoscopic Submucosal Dissection Training Using Thiel-Embalmed Cadavers

**DOI:** 10.1055/a-2904-9474

**Published:** 2026-07-23

**Authors:** Keiichi Hashiguchi, Maiko Tabuchi, Yuko Akazawa, Hiroko Inomata-Kawasaki, Junya Shiota, Taro Akashi, Moto Kitayama, Kayoko Matsushima, Naoyuki Yamaguchi, Ken Ohnita, Kazunobu Saiki, Keiko Ogami-Takamura, Hisamitsu Miyaaki

**Affiliations:** 1 Department of Endoscopy 88380 Nagasaki University HospitalNagasaki, Nagasaki PrefectureJapan; 2 Department of Gastroenterology and Hepatology 200674 Nagasaki University Graduate School of Biomedical SciencesNagasakiNagasaki PrefectureJapan; 3 Department of Histopathology and Cell Biology 200674 Nagasaki University Graduate School of Biomedical SciencesNagasakiNagasaki PrefectureJapan; 4 Medical Education Development Center 88380 Nagasaki University HospitalNagasakiNagasaki PrefectureJapan; 5 Department of Gastroenterology 157562 Inoue HospitalNagasakiNagasaki PrefectureJapan; 6 Department of Macroscopic Anatomy 200674 Nagasaki University Graduate School of Biomedical SciencesNagasaki, Nagasaki PrefectureJapan; 7 Center of Cadaver Surgical Training 643301 Nagasaki University School of MedicineNagasaki, Nagasaki PrefectureJapan

**Keywords:** cadaver surgical training, Thiel-embalmed cadaver, upper gastrointestinal endoscopy, endoscopic submucosal dissection

## Abstract

**Background and study aims** Duodenal endoscopic submucosal dissection (ESD) is technically challenging and associated with a high risk of adverse events. We evaluated duodenal ESD feasibility using cadaver surgical training (CST) with Thiel-embalmed cadavers.

**Patients and methods** Four Thiel-embalmed cadavers underwent upper gastrointestinal endoscopy, followed by simulated duodenal ESD. Seven endoscopists participated, including three experts and four trainees. The participants completed questionnaires evaluating procedural realism, technical difficulty, and educational value.

**Results** Upper gastrointestinal endoscopy was completed in all four cadavers, and duodenal ESD was conducted on seven simulated lesions. As occasionally encountered in clinical practice, the thin duodenal submucosal layer resulted in perforation in two lesions. The questionnaire responses demonstrated high median scores for procedural realism in upper gastrointestinal endoscopy and duodenal ESD. Participants agreed that CST closely replicated endoscopy in living patients and helped acquire duodenal ESD skills. Although psychological stress related to cadaver handling was reported, all participants expressed willingness to participate in future CST.

**Conclusions** Thiel-embalmed CST preserved the gastrointestinal mucosal architecture and was feasible for upper gastrointestinal endoscopy and duodenal ESD, suggesting its potential as a training model, warranting further validation with objective educational outcome measures.

## Introduction

Duodenal adenomas and adenocarcinomas that develop in regions of the duodenum other than the ampulla are referred to as superficial non-ampullary duodenal epithelial tumors (SNADETs). Their incidence has markedly increased in recent endoscopic practice. 1 2 Large SNADETs, akin to other gastrointestinal malignancies, should be resected en bloc via endoscopic submucosal dissection (ESD) because SNADETs may progress in accordance with an adenoma-carcinoma sequence. Nonetheless, the incidence of perforation associated with duodenal ESD is notably high (≥10%), often necessitating invasive interventions including pancreatoduodenectomy. 3 4 5

For gastric or colorectal ESD, appropriate training methods have involved animal models, nonanimal ex vivo models, and supervised clinical practice. 6 7 8 Currently, no suitable animal or simulation models have been reported for duodenal ESD. Therefore, endoscopists working in centers with lower case volumes must develop their expertise in duodenal ESD procedures through on-the-job training, with limited opportunities for skill enhancement and practice.

Cadaver surgical training (CST) is an educational method that uses human cadaver specimens to simulate realistic anatomic conditions and practice surgical procedures. CST is employed as an educational tool in general surgery and across various disciplines, including neurosurgery. 9 10 11 Furthermore, CST facilitates advanced applications such as research into novel techniques. 12 In CST, the Thiel-embalmed method is often used for fixation. 13 14 This method uses lower formalin concentrations (3–6%) compared to the conventional formalin method (8–10%) and includes propylene glycol and sodium sulfite. Consequently, the texture and flexibility of the skin, muscles, blood vessels, and nerves are preserved in a manner resembling that of a living organism. Accordingly, CST provides superior landmarks and tissue fidelity to simulation training for certain procedures. Nonetheless, CST use in gastrointestinal endoscopic treatment has been infrequently documented, with reports largely limited to common procedures such as colonoscopy insertion, endoscopic mucosal resection, and endoscopic variceal ligation. 15 16

Currently, no attempts have been made to employ CST for duodenal ESD. Consequently, this study aimed to evaluate the feasibility of duodenal ESD in Thiel-embalmed CST.

## Materials and Methods

### Cadavers and Participants

This single-center feasibility study analyzed a series of CST conducted at the Cadaver Surgical Training Center in Nagasaki, Japan, between July 2024 and February 2025. All four cadavers were donated by individuals. Informed consent was obtained from the donors and their families for use in medical education, scientific research, and CST. The cadavers were preserved using the Thiel-embalmed method and were confirmed to have no gastric contents by postmortem computed tomography. Upper gastrointestinal endoscopy (UGIE) was performed on each cadaver, followed by multiple duodenal ESD procedures.

Seven participants were recruited from the Department of Gastroenterology and Hepatology at Nagasaki University Hospital, Japan. Three participants had prior experience with duodenal ESD (expert group), whereas four were inexperienced (trainee group). Owing to the limited opportunities to perform duodenal ESD, this study categorized participants who had completed more than 100 cases of gastric and colorectal ESD and had participated in at least one duodenal ESD case as experts. Trainees were defined as those who had not performed duodenal ESD but had assisted in such procedures. Each participant engaged with at least one lesion and participated in all aspects of the duodenal ESD process, including marking, submucosal injection, circumferential incision, and submucosal dissection. The trainees were either supervised or assisted by experts throughout the procedure.

The study was conducted in accordance with the ethical guidelines of the Declaration of Helsinki (as revised in Fortaleza, Brazil, October 2013) and was approved by the Institutional Review Board of Nagasaki University Graduate School of Biomedical Sciences (approval no. 21052801-2).

### Endoscopic Device, Equipment, and Procedure

All endoscopic procedures were performed using a therapeutic gastroscope with a water-jet function (GIF-H290T; Olympus Medical Systems, Tokyo, Japan). UGIE was performed by a standard examination of the esophagus, stomach, and the descending part of the duodenum. For duodenal ESD, a tapered-tip hood (ST hood, DH-28GR; Fujifilm Corporation, Tokyo, Japan) or a straight hood (D-201-11804; Olympus Medical Systems, Tokyo, Japan) was attached to the endoscope tip. Multiple simulated lesions (10–15 mm) were created by marking in the second portion of the duodenum, followed by submucosal injection beneath each lesion. After a circumferential incision, submucosal dissection was performed to achieve en bloc excision. Most of the simulated lesions were created on the posterior wall of the descending duodenum, and when multiple lesions were present, care was taken to maintain adequate spacing to prevent interference. The water pressure method, 17 characterized by the application of active water pressure via the water-jet function, which facilitates expansion of the submucosa, thereby aiding the endoscope in accessing the area beneath the lesion and enabling submucosal dissection in confined spaces, was also employed. The injection solution comprised 0.4% sodium hyaluronate (MucoUp; Boston Scientific, Tokyo, Japan) with indigo carmine. For mucosal incision and submucosal dissection, a 1.5 mm needle-type knife (DualKnife J; Olympus Medical Systems, Tokyo, Japan, or FlushKnife BT-S; Fujifilm, Tokyo, Japan) was used. Additionally, the VIO 3 (ERBE Elektromedizin, Tübingen, Germany) device was used, employing Forced coagulation (effect 0.6) for marking, Endocut I (effect 1.0, duration 2.0, interval 2.0) for mucosal incisions, and precise SECT (effect 3.0) for submucosal dissection using the knife tip. Suturing of the mucosal defect and subsequent pathologic evaluation were not conducted because Ethics Committee approval was not obtained.

### Measured Outcomes

Participant questionnaires were administered to assess the feasibility of the procedure, simulation realism, and psychological barriers. UGIE evaluation criteria included endoscope insertion into the esophagus, duodenal shortening, insufflation, and suction in each segment, and examination of each segment. Duodenal ESD evaluation criteria included marking, submucosal injection, circumferential incision, submucosal dissection, and mucosal-architecture realism. The full questionnaire is presented in **Supplementary Table 1** . Each parameter was rated on a Likert scale (1 = strongly disagree, 2 = disagree, 3 = neutral, 4 = agree, 5 = strongly agree). Fidelity of technical difficulty (resemblance of technical challenges to living human conditions) was also assessed using a Likert scale (1 = significantly more challenging, 2 = more challenging, 3 = neutral, 4 = easy, 5 = significantly easy). Feasibility was defined as a median score greater than 4.0 on a 5-point Likert scale for each questionnaire. Among these, submucosal injection, circumferential incision, and submucosal dissection were identified as the most critical components. This prioritization is due to the primary objective of duodenal ESD in Thiel-embalmed CST: to replicate the complex scope maneuvering required in this anatomic region. Scores were compared between expert and trainee groups. Intention to participate in future CST was recorded as Yes, No, or Unknown.

### Statistical Analysis

The main analysis focused on descriptive statistics to assess the feasibility of CST. An exploratory intergroup comparison was conducted to inform the planning of future confirmatory trials. For the questionnaires, each score was aggregated and reported as the median score, along with the range. Wilcoxon rank-sum tests were used to compare quantitative variables, and *p* -values < 0.05 were considered statistically significant. All data analyses were performed using JMP Clinical version 17.2.0 (SAS Institute, Cary, NC, USA).

## Results



**Video 1** Endoscopic video of upper gastrointestinal endoscopy in cadaver surgical training.


**Video 2** Endoscopic video of duodenal endoscopic submucosal dissection in cadaver surgical training.


**Fig. 1:**
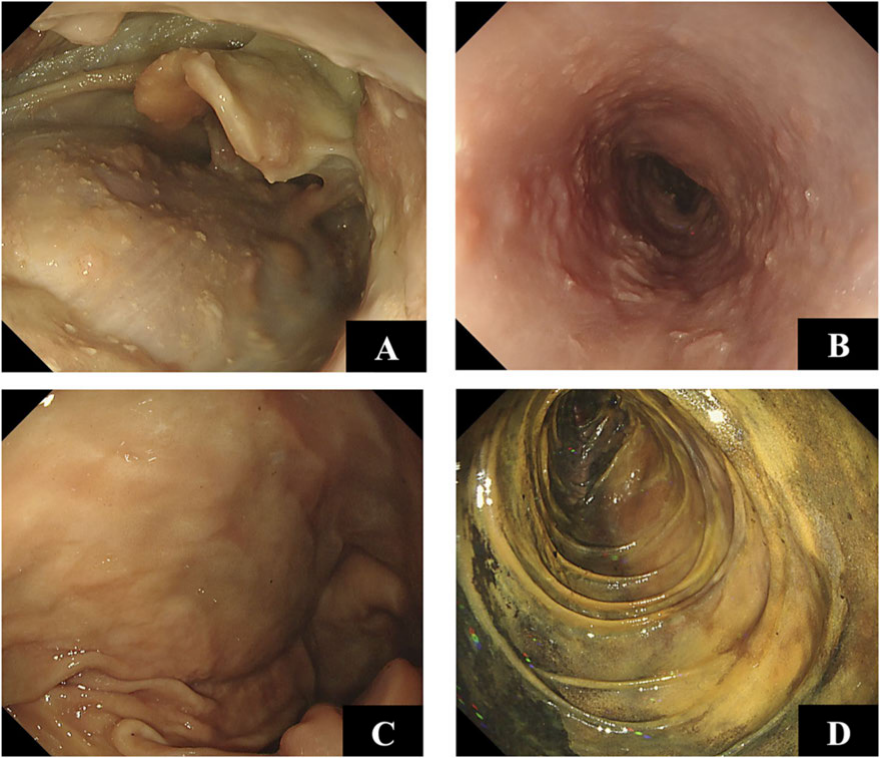
Endoscopic images of upper gastrointestinal endoscopy during cadaver surgical training. ( **A** ) Pharynx, ( **B** ) esophagus, ( **C** ) stomach, and ( **D** ) duodenum.

UGIE was completed in all four cadavers. A duodenal tear occurred in one case due to duodenal shortening; nonetheless, it did not affect subsequent procedures. Representative endoscopic images and a UGIE video are shown in Fig. 1 and Video 1 .

**Fig. 2:**
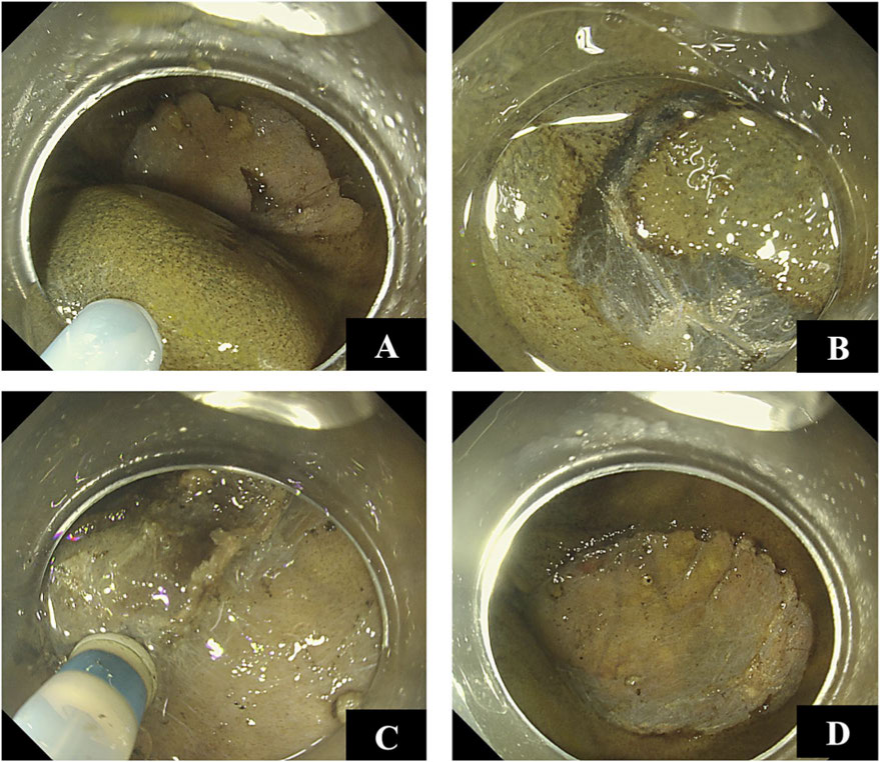
Endoscopic images of duodenal endoscopic submucosal dissection in cadaver surgical training. ( **A** ) Submucosal injection, ( **B** ) circumferential incision, ( **C** ) submucosal dissection, and ( **D** ) mucosal defect.

Duodenal ESD was performed on seven simulated lesions in four cadavers. As occasionally encountered in clinical practice, the duodenal submucosal layer was notably thin, resulting in perforations in two of the seven lesions. Representative endoscopic images and a video of the duodenal ESD are presented in Fig. 2 and Video 2 .

Table 1 presents survey findings on the perceived procedural realism of UGIE in CST and associated technical challenges. Median procedural scores (endoscopic insertion into the esophagus and observation at each segment) were predominantly high, rating 4 or 5 (range, 1–5), whereas intragastric observation scored 3 (range, 2–5).

**Table 1:** Survey findings concerning the realism and technical challenges of upper gastrointestinal endoscopy in cadaver surgical training.

Procedures	Overall ( *n* = 7)	Expert ( *n* = 3)	Trainee ( *n* = 4)	*p* -Values†
Esophagus				
Endoscopic insertion into the esophagus	4 (2–4)	4 (2–4)	3.5 (2–4)	1.000
Insufflation and suction	5 (4–5)	4 (4–5)	4.5 (4–5)	1.000
Examination	4 (3–5)	4 (3–5)	4.5 (3–5)	1.000
Stomach				
Insufflation and suction	4 (2–5)	4 (2–5)	4.5 (3–5)	0.657
Examination	3 (2–5)	3 (2–3)	4.5 (2–5)	0.229
Duodenum				
Insufflation and suction	4 (2–5)	4 (4)	4.5 (2–5)	0.771
Examination	5 (4–5)	5 (4–5)	5 (4–5)	1.000
Shortening	4 (1–5)	3 (1–4)	4 (3–5)	0.286

Score interpretation; 1 = significantly more challenging, 2 = more challenging, 3 = neutral, 4 = easy, 5 = significantly easy.

Scores are presented as medians (ranges).

†Expert group vs. trainee group.

Table 2 summarizes survey results on the perceived procedural realism and technical challenges of duodenal ESD in CST. Median procedural scores for key tasks, including submucosal injection and dissection, were consistently high (median 4; range 1–5). The item “Did you feel that the duodenal mucosal architecture was preserved during duodenal ESD in CST?” had a median score of 4.5 (range, 2–5). Table 3 presents findings on general inquiries and psychological impediments associated with CST. The item “Did you experience psychological stress while handling cadavers?” had a median score of 4 (range, 3–5). The items “Do you think CST could provide conditions similar to those of clinical endoscopy?” and “Do you think CST is useful for acquiring duodenal ESD technique?” had median scores of 4 (range, 3–5) and 4.5 (range, 2–5), respectively. The item “Do you think CST is superior to on-the-job training?” had a median score of 3.5 (range, 3–5). All respondents (100%) affirmed willingness to participate in CST again. No significant differences were observed between expert and trainee groups in any evaluation.

**Table 2:** Survey findings concerning the realism and technical challenges of duodenal endoscopic submucosal dissection in cadaver surgical training.

Procedures	Overall ( *n* = 7)	Expert ( *n* = 3)	Trainee ( *n* = 4)	*p* -Values†
Marking	4 (1–5)	4 (4)	5 (1–5)	0.400
Submucosal injection	4 (1–5)	4 (4–5)	4 (1–5)	1.000
Circumferential incision	4 (1–5)	4 (4–5)	4 (1–4)	0.600
Submucosal dissection	4 (1–4)	4 (3–4)	4 (1–4)	1.000
Mucosal architecture was preserved*	4.5 (2–5)	4 (4–5)	5 (2–5)	1.000

Score interpretation; 1 = significantly more challenging, 2 = more challenging, 3 = neutral, 4 = easy, 5 = significantly easy.

*Score interpretation; 1 = strongly disagree, 2 = disagree, 3 = neutral, 4 = agree, 5 = strongly agree.

Scores are presented as medians (ranges).

†Expert group vs. trainee group.

**Table 3:** Survey findings pertaining to general inquiries and psychological impediments associated with cadaver surgical training.

Queries	Overall ( *n* = 7)	Expert ( *n* = 3)	Trainee ( *n* = 4)	*p* -Values†
Did you experience psychological stress while handling the cadavers?	4 (3–5)	4 (3–5)	4 (3–4)	1.000
Do you think CST could provide conditions similar to those of clinical endoscopy?	4 (3–5)	4 (4–5)	4 (3–4)	0.600
Do you think CST is useful for acquiring the duodenal ESD technique?	4.5 (2–5)	5 (4–5)	4 (2–5)	0.700
Do you think CST is superior to on-the-job training?	3.5 (3–5)	5 (3–5)	3 (3–4)	0.400
Would you consider participating in future CST? Yes, *n* (%)	7 (100)	3 (100)	4 (100)	–

CST: cadaver surgical training, ESD: endoscopic submucosal dissection.

Score interpretation; 1 = strongly disagree, 2 = disagree, 3 = neutral, 4 = agree, 5 = strongly agree.

Scores are presented as medians (ranges).

†Expert group vs trainee group.

## Discussion

Here, we present the inaugural cases of duodenal ESD performed on a Thiel-embalmed CST. In the Thiel-embalmed CST, the gastrointestinal tract retained its flexibility and mucosal architecture, thereby facilitating duodenal ESD under conditions that closely mimic those in living patients. Therefore, CST has the potential to serve as a training model for duodenal ESD.

The CST is a historically validated gold-standard training tool for surgical residents, providing a high degree of realism in the technical manipulation and tactile sensation of human tissue, thereby facilitating early surgical independence. 9 18 Tominaga et al. identified the potential to enhance technical proficiency in advanced transanal surgery. 18 Nevertheless, the application of CST in gastrointestinal endoscopy remains limited due to the effectiveness of alternative training methods in this field. A multicenter study using virtual reality simulators demonstrated significant improvements in colonoscopy skills among surgical residents. 19 Another study found that more than 100 supervised procedures were required to achieve technical competence in UGIE and colonoscopy. 20 A multicenter comparative study reported significantly higher procedural speed in a group trained with a nonanimal gastric ESD model than in an untrained group, 21 supporting the roles of clinical experience and simulation-based training in endoscopic education. However, even endoscopists having substantial experience in ESD procedures outside the duodenum require 25 cases to achieve proficiency in duodenal ESD, whereas mastery is attained after 50 cases. 4 Furthermore, given the frequency and severity of complications, on-the-job training for duodenal ESD is inadvisable. No prior reports have described animal or simulation models for duodenal ESD. In our previous work, we described a duodenal ESD delayed perforation model utilizing in vivo porcine; however, owing to anatomic discrepancies (narrow duodenal lumen, narrow duodenal angle, etc.), it was unsuitable as a training model. 22 Given the technical complexity of duodenal ESD, attributable to the thin duodenal wall, limited submucosal working space, and unstable endoscope maneuverability, dedicated training opportunities are particularly important in this field. A realistic CST model may help endoscopists develop procedural familiarity and refine their technical skills in a controlled setting before performing these high-risk procedures in clinical practice. Whereas traditional formalin fixation causes tissue rigidity and reduced flexibility, the Thiel-embalmed method preserves tissue pliability. Given the limited use of CST in gastrointestinal endoscopic procedures, prior studies have documented only a narrow range of common procedures. Balekuduru et al. described a human cadaver hands-on training model for practicing endoscopic procedures, including percutaneous endoscopic gastrostomy, endoscopic variceal band ligation, endoscopic injection, snare polypectomy, thermal cautery, and endoscopic clip replacement. 15 Rohr et al. reported the effectiveness of reperfused human cadavers as a simulation model for colonoscopy. 16 Here, most responses indicated that the techniques were perceived as “easy” or “significantly easy” in the survey assessing the difficulty of each UGIE procedure. Specifically, this study further confirmed that insufflation and suction during UGIE can be performed analogously to procedures in living patients. Moreover, each duodenal ESD step was feasible, and CST reproduced limited endoscope maneuverability in the duodenum and the extremely thin mucosal architecture. For UGIE and duodenal ESD, no statistically significant differences were observed in scores for procedural difficulty or resemblance to living patients between expert and trainee groups. However, this study aimed to evaluate the feasibility of duodenal ESD on Thiel-embalmed CST, with intergroup comparisons conducted as exploratory analyses due to the limited sample size. Nonetheless, the observed data trends offer valuable insights for designing future confirmatory trials. Accordingly, Thiel-embalmed CST in endoscopic treatment may be suitable for low-frequency conditions and highly complex procedures, such as duodenal ESD. However, CST is costly and has limited availability compared with other training methods. 23 In Japanese medical schools, cadavers are donated for educational purposes. The estimated cost of preparing a Thiel-embalmed cadaver is approximately ¥40,000 per body; however, collaborative utilization is feasible in CST. By designating the anatomy department as a central hub, as is our practice, cadavers can be shared between the gastroenterology and surgical departments, mitigating economic burden and improving educational outcomes in multiple departments.

Nevertheless, CST has inherent limitations in endoscopic treatment. Unlike on-the-job training, it lacks respiratory fluctuations, peristalsis, and blood flow. Nonetheless, the same limitation is shared by many animal and simulation models. Importantly, CST requires collaboration with multiple departments, such as the Department of Anatomy, and provision of appropriate facilities and an adequate environment. Although survey responses in the present study indicated that cadaver handling may cause psychological stress, all participants expressed willingness to participate in future CST.

This study has some limitations. First, it was conducted at a single institution. Second, the number of CST sessions analyzed was small. Third, the educational effectiveness of CST has not yet been objectively verified. Future studies incorporating objective educational endpoints, such as procedure performance metrics, skill acquisition assessments, and comparative training outcomes, are necessary to establish its utility as a training platform.

In conclusion, this study presents the first application of duodenal ESD in CST. The Thiel-embalmed CST preserves the gastrointestinal mucosal architecture and is feasible for UGIE and duodenal ESD.

## Supplementary Material

Ergänzendes Material Supplementary Material
