# Supplementary material for: Feasibility of Duodenal Endoscopic Submucosal Dissection Training Using Thiel-Embalmed Cadavers
Source: Endosc Int Open. 2026 Jul 23;14:a29049474. doi: 10.1055/a-2904-9474 (PMC13463269; doi:10.1055/a-2904-9474)
Supplement: Supplementary file 1 [file 10-1055-a-2904-9474_29057429.pdf]

Supplementary material.

Author’s names: Keiichi Hashiguchi, Maiko Tabuchi, Yuko Akazawa, Hiroko Inomata, Junya Shiota, Taro Akashi, Moto Kitayama, Kayoko Matsushima, Naoyuki Yamaguchi, Ken Ohnita, Kazunobu Saiki, Keiko Ogami-Takamura, Hisamitsu Miyaaki

Title of paper: Feasibility of Duodenal Endoscopic Submucosal Dissection Training Using Thiel-Embalmed Cadavers

Supplementary table 1. Contents of the questionnaire

**Q1. How do you feel about each procedure of the upper gastrointestinal endoscopy in CST?**<br>**Please answer using the following scale for each procedure:**<br>◊ **1 = significantly more challenging**<br>◊ **2 = more challenging**<br>◊ **3 = neutral**<br>◊ **4 = easy**<br>◊ **5 = significantly easy**		
**Procedures**	Esophagus	Endoscopic insertion into the esophagus
		Insufflation and suction
		Examination
	Stomach	Insufflation and suction
		Examination
	Duodenum	Insufflation and suction
		Examination
		Shortening
**Q2-1. What is your opinion on each duodenal ESD procedure in the CST?**<br>**Please answer using the following scale for each procedure:**<br>◊ **1 = significantly more challenging**<br>◊ **2 = more challenging**<br>◊ **3 = neutral**<br>◊ **4 = easy**<br>◊ **5 = significantly easy**		

**Procedures**	Marking
	Submucosal injection
	Circumferential incision
	Submucosal dissection
**Q2-2. Did you feel that the duodenal mucosal architecture was preserved during duodenal ESD in CST?**<br>**Please answer using the following scale:**<br>◊ **1 = strongly disagree**<br>◊ **2 = disagree**<br>◊ **3 = neutral**<br>◊ **4 = agree**<br>◊ **5 = strongly agree**	
**Q3. What is your opinion about CST?**<br>**Please answer using the following scale for each question:**<br>◊ **1 = strongly disagree**<br>◊ **2 = disagree**<br>◊ **3 = neutral**<br>◊ **4 = agree**<br>◊ **5 = strongly agree**	
**Q3-1. Did you experience psychological stress while handling the cadavers?**	
**Q3-2. Do you think CST could provide conditions similar to those of clinical endoscopy?**	
**Q3-3. Do you think CST is useful for acquiring the duodenal ESD technique?**	
**Q3-4. Do you think CST is superior to on-the-job training?**	
**Q4. Would you consider participating in future CST?**<br>**Please answer using the following scale:**<br>◊ **Yes**<br>◊ **No**<br>◊ **Unknown**	

CST: cadaver surgical training, ESD: endoscopic submucosal dissection
